# Rule Acquisition in Formal Decision Contexts Based on Formal, Object-Oriented and Property-Oriented Concept Lattices

**DOI:** 10.1155/2014/685362

**Published:** 2014-08-03

**Authors:** Yue Ren, Jinhai Li, Cherukuri Aswani Kumar, Wenqi Liu

**Affiliations:** ^1^Faculty of Science, Kunming University of Science and Technology, Kunming, Yunnan 650500, China; ^2^School of Information Technology and Engineering, VIT University, Vellore 632014, India

## Abstract

Rule acquisition is one of the main purposes in the analysis of formal decision contexts. Up to now, there have been several types of rules in formal decision contexts such as decision rules, decision implications, and granular rules, which can be viewed as *∧*-rules since all of them have the following form: “if conditions 1,2,…, and *m* hold, then decisions hold.” In order to enrich the existing rule acquisition theory in formal decision contexts, this study puts forward two new types of rules which are called *∨*-rules and *∨*-*∧* mixed rules based on formal, object-oriented, and property-oriented concept lattices. Moreover, a comparison of *∨*-rules, *∨*-*∧* mixed rules, and *∧*-rules is made from the perspectives of inclusion and inference relationships. Finally, some real examples and numerical experiments are conducted to compare the proposed rule acquisition algorithms with the existing one in terms of the running efficiency.

## 1. Introduction

*Formal concept analysis* (FCA) is a field of applied mathematics based on the mathematization of* formal concepts* and conceptual hierarchy [1]. This theory starts with the notion of a* formal context *(*G*, *M*, *I*) consisting of an object set *G*, an attribute set *M*, and an incidence relation *I* between *G* and *M* [2]. Its key characteristic lies in the conceptual unfolding of data. Nowadays, FCA has been applied in many domains such as information retrieval [3], machine learning [4], knowledge discovery [5–15], and software engineering [16, 17]. What is more, it has shown a trend of multidisciplinary intersection.

In FCA, a basic way of describing dependencies between the attributes of a formal context is via* implications* [18, 19] or* association rules* [20]. Considering that directly deriving these types of rules from a formal context involves so many calculations and the number of rules is generally large, some researchers [21–23] discussed how to efficiently mine implications/association rules from a formal context and eliminate superfluous rules as many as possible. This issue was also investigated in generalized formal contexts [24].

In the real world, a formal context often contains target attributes for the purpose of making decision analysis. A formal context equipped with additional target attributes is called a* formal decision context* [25] (or a decision formal context sometimes) which is in fact a* training context* [26]. Note that rule acquisition is one of the main purposes in the analysis of formal decision contexts and in recent years this issue has attracted much attention from the Chinese FCA community. For instance, Zhang and Qiu [25] put forward the first type of rules, called* decision rules*, in formal decision contexts through combining conditional formal concepts with decision formal concepts. In order to eliminate superfluous decision rules, Li et al. [27] proposed the notion of a nonredundant decision rule and an approach to extract all nonredundant decision rules from a formal decision context, which was improved in [28] by integrating* granular computing* into decision rules for decreasing computation time. Moreover, the notion of a decision rule was extended into the cases of* incomplete formal decision contexts* [29] and* real formal decision contexts* [30, 31]. Besides, Qu et al. [32] presented the second type of rules, called* decision implications*, whose* premises* and* conclusions* are taken from conditional attributes and decision attributes, respectively. It should be pointed out that decision rules are special decision implications. Following the above discussion, Zhai et al. [33] discussed the semantical and syntactical aspects of decision implications. Additionally, based on granular computing, Wu et al. [34] defined the third type of rules, called* granular rules*, which are special decision rules, with their premises and conclusions being the intents of conditional formal concepts and decision formal concepts, respectively. A detailed investigation on relation between granular rules and decision rules can also be found in [28].

The aforementioned types of rules in formal decision contexts, which were investigated within the framework of classical FCA, can be viewed as ∧-rules since all of them have the following form: “if conditions 1,2,…, and *m* hold, then decisions hold.” Considering that object-oriented concept lattice [35] and property-oriented concept lattice [36] possess useful characteristics from both FCA and* rough set theory* [37] for data analysis and they have been proven to be beneficial to knowledge discovery [38, 39], this study puts forward two new types of rules, called ∨-rules and ∨-∧ mixed rules, using formal, object-oriented, and property-oriented concept lattices. The current work can enrich the existing rule acquisition theory in formal decision contexts.

The rest of this paper is organized as follows.  Section 2 reviews some basic notions and properties related to formal, object-oriented, and property-oriented concept lattices.  Section 3 discusses the issue of rule acquisition in formal decision contexts based on formal, object-oriented, and property-oriented concept lattices. More specifically, the notions of a ∨-rule and a ∨-∧ mixed rule are proposed. Furthermore, we put forward approaches to derive all nonredundant ∨-rules and ∨-∧ mixed rules from a formal decision context.  Section 4 makes a comparison of the inclusion and inference relationships among ∨-rules, ∨-∧ mixed rules, and ∧-rules.  Section 5 conducts some numerical experiments to compare the performances of the proposed rule acquisition algorithms and the existing one.

## 2. Preliminaries

In what follows, we briefly recall some basic notions and properties related to formal, object-oriented, and property-oriented concept lattices.

**Definition 1 (see [1]).**

A formal context is a triple (*G*, *M*, *I*) including an object set *G*, an attribute set *M*, and an incidence relation *I*⊆*G* × *M*, in which (*x*, *a*) ∈ *I* indicates that the object *x* has the attribute *a* and (*x*, *a*) ∉ *I* means the opposite.

**Definition 2 (see [25]).**

A formal context (*G*, *M*, *I*) is said to be regular if, for any (*x*, *a*) ∈ *G* × *M*, the following conditions hold:

- there exist *a*_1_, *a*_2_ ∈ *M* such that (*x*, *a*_1_) ∈ *I* and (*x*, *a*_2_) ∉ *I*;
- there exist *x*_1_, *x*_2_ ∈ *G* such that (*x*_1_, *a*) ∈ *I* and (*x*_2_, *a*) ∉ *I*.

Without loss of generality, the formal contexts discussed hereinafter are all assumed to be regular.

In order to derive formal, object-oriented, and property-oriented concept lattices, the following six operators are needed: for any *X*⊆*G* and *B*⊆*M*,

(1) $$\begin{array}{c} X^{↑}=\left\{a\in M\;|\;\;\text{for}\;\text{all}\;x\in X,\left(x,a\right)\in I\right\},\\ X^{□}=\left\{a\in M\;|\;\text{for}\;\text{all}\;x\in G,\text{if}\;\left(x,a\right)\in I,\text{then}\;x\in X\right\},\\ X^{⋄}=\left\{a\in M\;|\;\text{there}\;\text{exists}\;x\in X\;\text{such}\;\text{that}\;\left(x,a\right)\in I\right\},\\ B^{↓}=\left\{x\in G\;|\;\text{for}\;\text{all}\;a\in B,\left(x,a\right)\in I\right\},\\ B^{□}=\left\{x\in G\;|\;\text{for}\;\text{all}\;a\in M,\text{if}\;\left(x,a\right)\in I,a\in B\right\},\\ B^{⋄}=\left\{x\in G\;|\;\;\text{there}\;\text{exists}\;a\in B\;\text{such}\;\text{that}\;\left(x,a\right)\in I\right\}. \end{array}$$

Note that the pair of operators (↑, ↓) forms an antitone Galois connection, while the pairs of operators (□, ⋄) and (⋄, □) form isotone Galois connections [39]. More properties about these operators can be found below.

**Proposition 3 (see [2, 35, 36]).**

Let (*G*, *M*, *I*) be a formal context. For *X*, *X*_1_, *X*_2_⊆*G* and *B*, *B*_1_, *B*_2_⊆*M*, the following properties hold:

- *X*_1_⊆*X*_2_⇒*X*_2_^↑^⊆*X*_1_^↑^, *X*_1_^□^⊆*X*_2_^□^, *X*_1_^⋄^⊆*X*_2_^⋄^;
- *B*_1_⊆*B*_2_⇒*B*_2_^↓^⊆*B*_1_^↓^, *B*_1_^□^⊆*B*_2_^□^, *B*_1_^⋄^⊆*B*_2_^⋄^;
- *X*⊆*X* ^↑↓^, *X* ^□⋄^⊆*X*⊆*X* ^⋄□^;
- *B*⊆*B* ^↓↑^, *B* ^□⋄^⊆*B*⊆*B* ^⋄□^;
- *X* ^↑↓↑^ = *X* ^↑^, *X* ^□⋄□^ = *X* ^□^, *X* ^⋄□⋄^ = *X* ^⋄^;
- *B* ^↓↑↓^ = *B* ^↓^, *B* ^□⋄□^ = *B* ^□^, *B* ^⋄□⋄^ = *B* ^⋄^.

**Definition 4 (see [1, 2, 35, 36]).**

Let (*G*, *M*, *I*) be a formal context, *X*⊆*G*, and *B*⊆*M*. If *X* ^↑^ = *B* and *B* ^↓^ = *X*, then (*X*, *B*) is called a formal concept; if *X* ^□^ = *B* and *B* ^⋄^ = *X*, then (*X*, *B*) is called an object-oriented concept; if *X* ^⋄^ = *B* and *B* ^□^ = *X*, then (*X*, *B*) is called a property-oriented concept. For each of the cases, *X* and *B* are called the extent and intent of (*X*, *B*), respectively.

When the formal, object-oriented, and property-oriented concepts of a formal context (*G*, *M*, *I*) are, respectively, ordered by

(2) $$\begin{array}{c} \left(X_{1},B_{1}\right)\leq_{F}\left(X_{2},B_{2}\right)⟺X_{1}\subseteq X_{2}\;\left(⟺B_{2}\subseteq B_{1}\right),\\ \left(X_{1},B_{1}\right)\leq_{O}\left(X_{2},B_{2}\right)⟺X_{1}\subseteq X_{2}\;\left(⟺B_{1}\subseteq B_{2}\right),\\ \left(X_{1},B_{1}\right)\leq_{P}\left(X_{2},B_{2}\right)⟺X_{1}\subseteq X_{2}\;\left(⟺B_{1}\subseteq B_{2}\right), \end{array}$$

they form complete lattices which are called the formal, object-oriented, and property-oriented concept lattices [1, 35, 36] of the formal context (*G*, *M*, *I*), respectively. Hereinafter, we denote formal concept lattice by ${\underline{B}}_{F}(G,M,I)$, object-oriented concept lattice by ${\underline{B}}_{O}(G,M,I)$, and property-oriented concept lattice by ${\underline{B}}_{P}(G,M,I)$.

In the concept lattices ${\underline{B}}_{F}(G,M,I)$, ${\underline{B}}_{O}(G,M,I)$, and ${\underline{B}}_{P}(G,M,I)$, the* infimum* and* supremum* of two concepts (*X*_1_, *B*_1_) and (*X*_2_, *B*_2_) are, respectively, defined by

(3) $$\begin{array}{c} \left(X_{1},B_{1}\right)\wedge_{F}\left(X_{2},B_{2}\right)=\left(X_{1}\cap X_{2},{\left(B_{1}\cup B_{2}\right)}^{↓↑}\right),\\ \left(X_{1},B_{1}\right)\vee_{F}\left(X_{2},B_{2}\right)=\left({\left(X_{1}\cup X_{2}\right)}^{↑↓},B_{1}\cap B_{2}\right);\\ \left(X_{1},B_{1}\right)\wedge_{O}\left(X_{2},B_{2}\right)=\left({\left(X_{1}\cap X_{2}\right)}^{□⋄},B_{1}\cap B_{2}\right),\\ \left(X_{1},B_{1}\right)\vee_{O}\left(X_{2},B_{2}\right)=\left(X_{1}\cup X_{2},{\left(B_{1}\cup B_{2}\right)}^{⋄□}\right);\\ \left(X_{1},B_{1}\right)\wedge_{P}\left(X_{2},B_{2}\right)=\left(X_{1}\cap X_{2},{\left(B_{1}\cap B_{2}\right)}^{□⋄}\right),\\ \left(X_{1},B_{1}\right)\vee_{P}\left(X_{2},B_{2}\right)=\left({\left(X_{1}\cup X_{2}\right)}^{⋄□},B_{1}\cup B_{2}\right). \end{array}$$

It should be pointed out that the relation among formal, object-oriented, and property-oriented concept lattices was discussed in [38, 39], and knowledge reduction of object-oriented and/or property-oriented concept lattices was investigated in [7, 40, 41].

## 3. Rule Acquisition in Formal Decision Contexts Based on Formal, Object-Oriented, and Property-Oriented Concept Lattices

**Definition 5 (see [25, 26]).**

A formal decision context is a quintuple (*G*, *M*, *I*, *N*, *J*), where (*G*, *M*, *I*) and (*G*, *N*, *J*) with *M*∩*N* = *∅* are two formal contexts. Here, *M* and *N* are called the conditional attribute set and the decision attribute set of (*G*, *M*, *I*, *N*, *J*), respectively.

Like the formal context, a formal decision context Π = (*G*, *M*, *I*, *N*, *J*) is also said to be regular [27] if both (*G*, *M*, *I*) and (*G*, *N*, *J*) are regular. Hereinafter, the concerned formal decision contexts are all assumed to be regular. Moreover, for convenience, ${\underline{B}}_{F}(G,M,I)$, ${\underline{B}}_{O}(G,M,I)$, and ${\underline{B}}_{P}(G,M,I)$ are, respectively, called conditional formal, object-oriented, and property-oriented concept lattices of Π, and ${\underline{B}}_{F}(G,N,J)$,${\underline{B}}_{O}(G,N,J)$, and ${\underline{B}}_{P}(G,N,J)$ are, respectively, called decision formal, object-oriented, and property-oriented concept lattices of Π.

To the best of our knowledge, the existing work of rule acquisition in formal decision contexts is based on (conditional and decision) formal concept lattices only, and the derived rules *B* → *C* with *B*⊆*M* and *C*⊆*N* can be viewed as ∧-ones since they have the following form: “any object having all conditional attributes of *B* also has all decision attributes of *C*.” Up to now, such kinds of ∧-rules have successfully been applied to radar fault diagnosis under incomplete environment [42].

In order to widen the domain of application of rule acquisition, now we continue to put forward two new types of rules, called ∨-rules and ∨-∧ mixed rules, based on formal, object-oriented, and property-oriented concept lattices.

### 3.1. Rule Acquisition Based on Formal and Object-Oriented Concept Lattices

In this subsection, we propose the notion of a ∨-rule in formal decision contexts based on formal and object-oriented concept lattices.

**Definition 6 .**

Let Π = (*G*, *M*, *I*, *N*, *J*) be a formal decision context, let ${\underline{B}}_{O}(G,M,I)$ be the object-oriented concept lattice of (*G*, *M*, *I*), and let ${\underline{B}}_{F}(G,N,J)$ be the formal concept lattice of (*G*, *N*, *J*). For any $\left(X,B)\in {\underline{B}}_{O}(G,M,I\right)$ and $\left(Y,C)\in {\underline{B}}_{F}(G,N,J\right)$, if *X* ≠ *∅*, *Y* ≠ *G*, and *X*⊆*Y*, then the expression *B*→_∨_ *C* is called a ∨-rule generated between the object-oriented concept (*X*, *B*) and formal concept (*Y*, *C*). Here, *B* and *C* are called the premise and conclusion of the ∨-rule *B* → *C*, respectively. The set of all the ∨-rules generated between the object-oriented concepts in ${\underline{B}}_{O}(G,M,I)$ and the formal concepts in ${\underline{B}}_{F}(G,N,J)$ is denoted by *R*_*O*_(Π).

Thus, for any *B*→_∨_ *C* ∈ *R*_*O*_(Π), we conclude that each *x* ∈ *G* having at least one conditional attribute in *B* has all the decision attributes in *C*. More specifically, if *B* = {*b*_1_, *b*_2_,…, *b*_*s*_} and *C* = {*c*_1_, *c*_2_,…, *c*_*t*_}, then *B*→_∨_ *C* means that “if *b*_1_∨*b*_2_∨⋯∨*b*_*s*_, then *c*_1_∧*c*_2_∧⋯∧*c*_*t*_,” where ∨ and ∧ denote logical disjunction and conjunction operators.

It should be pointed out that the ∨-rules have something to do with both the attribute implication rules and the association rules (see, e.g., [18, 19] for the detailed introduction of the attribute implication rules and, e.g., [20, 23] for that of the association rules). Concretely, a ∨-rule *B*→_∨_ *C* with *B* = {*b*_1_, *b*_2_,…, *b*_*s*_} and *C* = {*c*_1_, *c*_2_,…, *c*_*t*_} can be integrated by the following attribute implication rules (or association rules with their confidences being one):

(4) b1⟶c1∧c2∧⋯∧ct,b2⟶c1∧c2∧⋯∧ct,  ⋮bs⟶c1∧c2∧⋯∧ct.

However, an attribute implication rule may not be a ∨-rule since its premise is not an expression of disjunction of conditional attributes except a singleton set. Yet, an association rule may not be a ∨-rule since its confidence is often less than one.

**Definition 7 .**

Let Π = (*G*, *M*, *I*, *N*, *J*) be a formal decision context. For *B*_1_→_∨_ *C*_1_, *B*_2_→_∨_ *C*_2_ ∈ *R*_*O*_(Π), if *B*_2_⊆*B*_1_ and *C*_2_⊆*C*_1_, one says that *B*_2_→_∨_ *C*_2_ can be implied by *B*_1_→_∨_ *C*_1_. One denotes this implication relationship by *B*_1_→_∨_ *C*_1_⇒*B*_2_→_∨_ *C*_2_. For any *B*→_∨_ *C* ∈ *R*_*O*_(Π), if there exists *B*_0_→_∨_ *C*_0_ ∈ *R*_*O*_(Π)∖{*B*→_∨_ *C*} such that *B*_0_→_∨_ *C*_0_⇒*B*→_∨_ *C*, then *B*→_∨_ *C* is said to be redundant in *R*_*O*_(Π); otherwise, *B*→_∨_ *C* is said to be nonredundant in *R*_*O*_(Π). We denote by *R*_O_*(Π) the set of all of the nonredundant ∨-rules in *R*_*O*_(Π).

It can be known from Definition 7 that, for a given formal decision context, it is more appealing to extract the nonredundant ∨-rules since they can imply others.

Let Π = (*G*, *M*, *I*, *N*, *J*) be a formal decision context. Denote

(5) $$\begin{array}{c} {\underline{U}}_{O}\left(G,M,I\right)=\left\{X\;|\;\left(X,B\right)\in {\underline{B}}_{O}\left(G,M,I\right)\right\},\\ {\underline{U}}_{F}\left(G,N,J\right)=\left\{Y\;|\;\left(Y,C\right)\in {\underline{B}}_{F}\left(G,N,J\right)\right\}. \end{array}$$

For any $\left(X,Y)\in {\underline{U}}_{O}(G,M,I)\times {\underline{U}}_{F}(G,N,J\right)$, define

(6) αO(X,Y)={1,if  X⊆Y  and  there  does  not  exist  X0 ∈U_O(G,M,I)  such  that  X⊂X0⊆Y,0,otherwise,βO(X,Y)={1,if  X⊆Y  and  there  does  not  exist  Y0 ∈U_F(G,N,J)  such  that  X⊆Y0⊂Y,0,otherwise.

**Theorem 8 .**

For a formal decision context Π = (*G*, *M*, *I*, *N*, *J*), $\left(X,B)\in {\underline{B}}_{O}(G,M,I\right)$, $\left(Y,C)\in {\underline{B}}_{F}(G,N,J\right)$, and *B*→_∨_ *C* ∈ *R*_*O*_(Π). Then, *B*→_∨_ *C* is redundant in *R*_*O*_(Π) if and only if *α*_*O*_(*X*, *Y*) = 0 or *β*_*O*_(*X*, *Y*) = 0, or, equivalently, *B*→_∨_ *C* is nonredundant in *R*_*O*_(Π) if and only if *α*_*O*_(*X*, *Y*) = 1 and *β*_*O*_(*X*, *Y*) = 1.

**Proof**

Necessity. If *B*→_∨_ *C* is redundant in *R*_*O*_(Π), there exists *B*_0_→_∨_ *C*_0_ ∈ *R*_*O*_(Π)∖{*B*→_∨_ *C*} such that $\left(X_{0},B_{0})\in {\underline{B}}_{O}(G,M,I\right)$, $\left(Y_{0},C_{0}\right)\in {\underline{B}}_{F}\left(G,N,J\right)$, and *B*_0_→_∨_ *C*_0_⇒*B*→_∨_ *C*. By Definition 7, it follows that *B*⊆*B*_0_ and *C*⊆*C*_0_, which implies that *X*⊆*X*_0_ and *Y*_0_⊆*Y*. Noting that *X*_0_⊆*Y*_0_ and *B*_0_→_∨_ *C*_0_ is different from *B*→_∨_ *C*, we conclude that *X* ⊂ *X*_0_⊆*Y*_0_⊆*Y* or *X*⊆*X*_0_⊆*Y*_0_ ⊂ *Y*. Consequently, *α*_*O*_(*X*, *Y*) = 0 or *β*_*O*_(*X*, *Y*) = 0.

Sufficiency. If *α*_*O*_(*X*, *Y*) = 0 or *β*_*O*_(*X*, *Y*) = 0, we can prove that *B*→_∨_ *C* is redundant in *R*_*O*_(Π). In fact, when *α*_*O*_(*X*, *Y*) = 0, there exists $X_{0}\in {\underline{U}}_{O}(G,M,I)$ such that *X* ⊂ *X*_0_⊆*Y*. Suppose $\left(X_{0},B_{0})\in {\underline{B}}_{O}(G,M,I\right)$. Then, *B*_0_→_∨_ *C*⇒*B*→_∨_ *C*. As a result, *B*→_∨_ *C* is redundant in *R*_*O*_(Π). When *β*_*O*_(*X*, *Y*) = 0, there exists $Y_{0}\in {\underline{U}}_{F}(G,N,J)$ such that *X*⊆*Y*_0_ ⊂ *Y*. Suppose $\left(Y_{0},C_{0})\in {\underline{B}}_{F}(G,N,J\right)$. Then, *B*→_∨_ *C*_0_⇒*B*→_∨_ *C*. Consequently, *B*→_∨_ *C* is redundant in *R*_*O*_(Π).

Now, we are ready to put forward a method to derive the nonredundant ∨-rules from a formal decision context. The method can briefly be described as in Algorithm 1.

Note that the object-oriented concept lattice of (*G*, *M*, *I*) can be derived from the formal concept lattice of the complementary formal context of (*G*, *M*, *I*) [39]. Then, it is easy to prove that the time complexity of Algorithm 1 is

(7) O((|G|+|M|)|M||LO|+(|G|+|N|)|N||LF|  +|G||LO|2|LF|+|G||LO||LF|2),

where |*L*_*O*_| denotes the cardinality of the object-oriented concept lattice of (*G*, *M*, *I*) and |*L*_*F*_| denotes that of the formal concept lattice of (*G*, *N*, *J*).

**Example 9 .**

**Figure 1:**
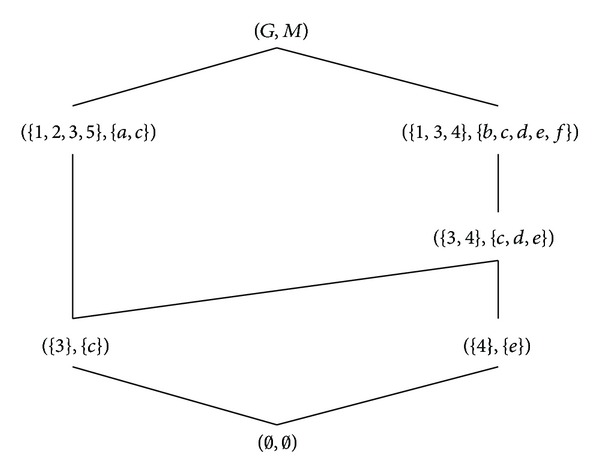
The Hasse diagram of ${\underline{B}}_{O}(G,M,I)$.

**Figure 2:**
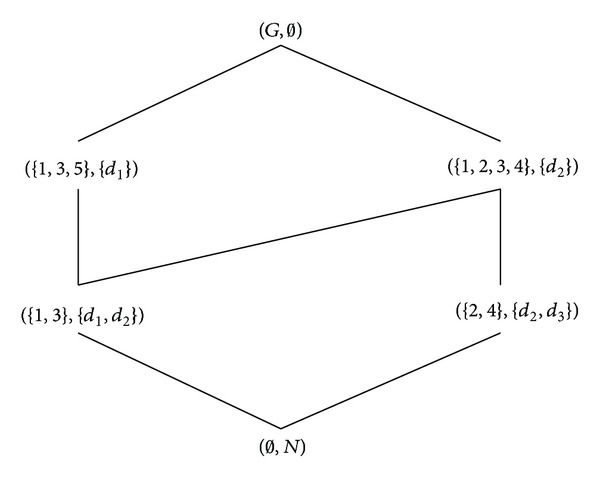
The Hasse diagram of ${\underline{B}}_{F}(G,N,J)$.

Table 1 depicts a formal decision context Π = (*G*, *M*, *I*, *N*, *J*), where *G* = {1,2, 3,4, 5}, *M* = {*a*, *b*, *c*, *d*, *e*, *f*}, and *N* = {*d*_1_, *d*_2_, *d*_3_}. The object-oriented concept lattice of (*G*, *M*, *I*) is shown in Figure 1 and the formal concept lattice of (*G*, *N*, *J*) is shown in Figure 2.

According to Algorithm 1, we can derive the following nonredundant ∨-rules from Π:

 
*r*_1_: if *c*, then *d*_1_ and *d*_2_;
 
*r*_2_: if *e*, then *d*_2_ and *d*_3_;
 
*r*_3_: if *b*, *c*, *d*, *e*, or *f*, then *d*_2_.

It should be pointed out that *r*_3_ can be divided into the following attribute implication rules:

 
*r*_3(1)_: if *b*, then *d*_2_;
 
*r*_3(2)_: if *c*, then *d*_2_;
 
*r*_3(3)_: if *d*, then *d*_2_;
 
*r*_3(4)_: if *e*, then *d*_2_;
 
*r*_3(5)_: if *f*, then *d*_2_.

### 3.2. Rule Acquisition Based on Formal and Property-Oriented Concept Lattices

In this subsection, we continue to put forward the notion of a ∨-∧ mixed rule in formal decision contexts based on formal and property-oriented concept lattices.

**Definition 10 .**

Let Π = (*G*, *M*, *I*, *N*, *J*) be a formal decision context, let ${\underline{B}}_{P}(G,M,I)$ be the property-oriented concept lattice of (*G*, *M*, *I*), and let ${\underline{B}}_{F}(G,N,J)$ be the formal concept lattice of (*G*, *N*, *J*). For any $\left(X,B)\in {\underline{B}}_{P}(G,M,I\right)$ and $\left(Y,C)\in {\underline{B}}_{F}(G,N,J\right)$, if *X* ≠ *∅*, *Y* ≠ *G*, and *X*⊆*Y*, then the expression *B*→_∨∧_ *C* is called a ∨-∧ mixed rule generated between the property-oriented concept (*X*, *B*) and the formal concept (*Y*, *C*). Here, *B* and *C* are called the premise and conclusion of the ∨-∧ mixed rule *B* → *C*, respectively. The set of all of the ∨-∧ mixed rules generated between the property-oriented concepts in ${\underline{B}}_{P}(G,M,I)$ and the formal concepts in ${\underline{B}}_{F}(G,N,J)$ is denoted by *R*_*P*_(Π).

Thus, for any *B*→_∨∧_ *C* ∈ *R*_*P*_(Π), we conclude that each object having at least one conditional attribute in *B* and no conditional attribute in *M*∖*B* has all the decision attributes in *C*. More specifically, if *B* = {*b*_1_, *b*_2_,…, *b*_*s*_}, *M*∖*B* = {*b*_*s*+1_, *b*_*s*+2_,…, *b*_*n*_}, and *C* = {*c*_1_, *c*_2_,…, *c*_*t*_}, then *B*→_∨∧_ *C* means the following: “if *b*_1_  ∨*b*_2_  ∨⋯∨  *b*_*s*_ and ¬*b*_*s*+1_  ∧  ¬*b*_*s*+2_∧  ⋯  ∧  ¬*b*_*n*_, then *c*_1_  ∧  *c*_2_  ∧  ⋯  ∧  *c*_*t*_,” where ∨, ∧, and ¬ denote logical disjunction, conjunction, and negation operators, respectively.

It should be pointed out that the ∨-∧ mixed rules have something to do with both the attribute implication rules and the association rules. Concretely, a ∨-∧ mixed rule *B*→_∨∧_ *C* with *B* = {*b*_1_, *b*_2_,…, *b*_*s*_}, *M*∖*B* = {*b*_*s*+1_, *b*_*s*+2_,…, *b*_*n*_}, and *C* = {*c*_1_, *c*_2_,…, *c*_*t*_} can be integrated by the following attribute implication rules (or association rules with their confidences being one):

(8) b1∧¬bs+1∧¬bs+2∧⋯∧¬bn⟶c1∧c2∧⋯∧ct,b2∧¬bs+1∧¬bs+2∧⋯∧¬bn⟶c1∧c2∧⋯∧ct,  ⋮bs∧¬bs+1∧¬bs+2∧⋯∧¬bn⟶c1∧c2∧⋯∧ct.

However, an attribute implication rule may not be a ∨-∧ mixed rule since its premise is generally not an expression of disjunction and conjunction of conditional attributes. Yet, an association rule may not be a ∨-∧ mixed rule since its confidence is often less than one.

**Definition 11 .**

Let Π = (*G*, *M*, *I*, *N*, *J*) be a formal decision context. For any *B*_1_→_∨∧_ *C*_1_, *B*_2_→_∨∧_ *C*_2_ ∈ *R*_*P*_(Π), if *B*_2_⊆*B*_1_ and *C*_2_⊆*C*_1_, one says that *B*_2_→_∨∧_ *C*_2_ can be implied by *B*_1_→_∨∧_ *C*_1_. One denotes this implication relationship by *B*_1_→_∨∧_ *C*_1_⇒*B*_2_→_∨∧_ *C*_2_. For any *B*→_∨∧_ *C* ∈ *R*_*P*_(Π), if there exists *B*_0_→_∨∧_ *C*_0_ ∈ *R*_*P*_(Π)∖{*B*→_∨∧_ *C*} such that *B*_0_→_∨∧_ *C*_0_⇒*B*→_∨∧_ *C*, then *B*→_∨∧_ *C* is said to be redundant in *R*_*P*_(Π); otherwise, *B*→_∨∧_ *C* is said to be nonredundant in *R*_*P*_(Π). One denotes by *R*_P_*(Π) the set of all the nonredundant ∨-∧ mixed rules in *R*_*P*_(Π).

It can be known from Definition 11 that, for a given formal decision context, it is more appealing to extract the nonredundant ∨-∧ mixed rules since they can imply the remainder.

Let Π = (*G*, *M*, *I*, *N*, *J*) be a formal decision context. Denote

(9) U_P(G,M,I)={X ∣ (X,B)∈B_P(G,M,I)},U_F(G,N,J)={Y ∣ (Y,C)∈B_F(G,N,J)}.

For any $\left(X,Y)\in {\underline{U}}_{P}(G,M,I)\times {\underline{U}}_{F}(G,N,J\right)$, one defines

(10) αP(X,Y)={1,if  X⊆Y  and  there  does  not  exist  X0 ∈U_P(G,M,I)  such  that  X⊂X0⊆Y,0,otherwise,βP(X,Y)={1,if  X⊆Y  and  there  does  not  exist  Y0 ∈U_F(G,N,J)  such  that  X⊆Y0⊂Y,0,otherwise.

**Theorem 12 .**

For a formal decision context Π = (*G*, *M*, *I*, *N*, *J*), $\left(X,B)\in {\underline{B}}_{P}(G,M,I\right)$, $\left(Y,C)\in {\underline{B}}_{F}(G,N,J\right)$, and *B*→_∨∧_ *C* ∈ *R*_*P*_(Π). Then, *B*→_∨∧_ *C* is redundant in *R*_*P*_(Π) if and only if *α*_*P*_(*X*, *Y*) = 0 or *β*_*P*_(*X*, *Y*) = 0, or, equivalently, *B*→_∨∧_ *C* is nonredundant in *R*_*P*_(Π) if and only if *α*_*P*_(*X*, *Y*) = 1 and *β*_*P*_(*X*, *Y*) = 1.

**Proof**

It is similar to the proof of Theorem 8.

Now, we are ready to propose an approach to derive all the nonredundant ∨-∧ mixed rules from a formal decision context. The detailed steps are given in Algorithm 2.

Note that the property-oriented concept lattice of (*G*, *M*, *I*) can be derived from the formal concept lattice of the complementary formal context of (*G*, *M*, *I*) [39]. Then, it is easy to prove that the time complexity of Algorithm 2 is

(11) O((|G|+|M|)|M||LP|+(|G|+|N|)|N||LF|  +|G||LP|2|LF|+|G||LP||LF|2),

where |*L*_*P*_| denotes the cardinality of the property-oriented concept lattice of (*G*, *M*, *I*) and |*L*_*F*_| denotes that of the formal concept lattice of (*G*, *N*, *J*).

**Example 13 .**

**Figure 3:**
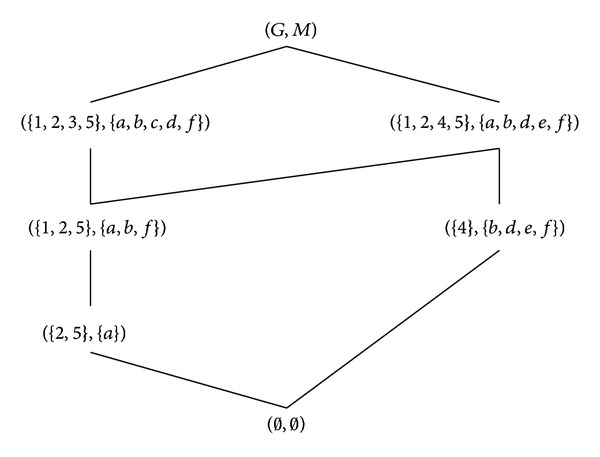
The Hasse diagram of ${\underline{B}}_{P}(G,M,I)$.

Let Π = (*G*, *M*, *I*, *N*, *J*) be the formal decision context in Table 1, where *G* = {1,2, 3,4, 5}, *M* = {*a*, *b*, *c*, *d*, *e*, *f*}, and *N* = {*d*_1_, *d*_2_, *d*_3_}. The property-oriented concept lattice of (*G*, *M*, *I*) is shown in Figure 3 and the formal concept lattice of (*G*, *N*, *J*) can be found in Figure 2.

According to Algorithm 2, we can derive the following nonredundant ∨-∧ mixed rule from Π:

 
*r*′: if *b*, *d*, *e*, or *f* and ¬*a* and ¬*c*, then *d*_2_ and *d*_3_.

It should be pointed out that *r*′ can be divided into the following attribute implication rules:

 
*r*_1_′: if *b*, ¬*a*, and ¬*c*, then *d*_2_ and *d*_3_;
 
*r*_2_′: if *d*, ¬*a*, and ¬*c*, then *d*_2_ and *d*_3_;
 
*r*_3_′: if *e*, ¬*a*, and ¬*c*, then *d*_2_ and *d*_3_;
 
*r*_4_′: if *f*, ¬*a*, and ¬*c*, then *d*_2_ and *d*_3_.

## 4. A Comparison of Rules in Terms of Inclusion and Inference Relationships

In Section 3, we have compared the ∨-rules and ∨-∧ mixed rules with the attribute implication rules and association rules. In this section, we continue to make a comparison of ∨-rules, ∨-∧ mixed rules, and decision rules in terms of inclusion and inference relationships. Before embarking on this issue, we introduce the notion of a decision rule in formal decision contexts.

**Definition 14 (see [27]).**

Let Π = (*G*, *M*, *I*, *N*, *J*) be a formal decision context, let ${\underline{B}}_{F}(G,M,I)$ be the formal concept lattice of (*G*, *M*, *J*), and let ${\underline{B}}_{F}(G,N,J)$ be the formal concept lattice of (*G*, *N*, *J*). For any $\left(X,B)\in {\underline{B}}_{F}(G,M,I\right)$ and $\left(Y,C)\in {\underline{B}}_{F}(G,N,J\right)$, if *X*, *B*, *Y*, and *C* are all nonempty and *X*⊆*Y*, then the expression *B*→_∧_ *C* is called a decision rule generated between the formal concepts (*X*, *B*) and (*Y*, *C*). Here, *B* and *C* are called the premise and conclusion of the decision rule *B*→_∧_ *C*, respectively. The set of all the decision rules generated between the formal concepts in ${\underline{B}}_{F}(G,M,I)$ and those in ${\underline{B}}_{F}(G,N,J)$ is denoted by *R*_*F*_(Π).

**Definition 15 (see [27]).**

Let Π = (*G*, *M*, *I*, *N*, *J*) be a formal decision context. For *B*_1_→_∧_ *C*_1_, *B*_2_→_∧_ *C*_2_ ∈ *R*_*F*_(Π), if *B*_1_⊆*B*_2_ and *C*_2_⊆*C*_1_, one says that *B*_2_→_∧_ *C*_2_ can be implied by *B*_1_→_∧_ *C*_1_. One denotes this implication relationship by *B*_1_→_∧_ *C*_1_⇒*B*_2_→_∧_ *C*_2_. For any *B*→_∧_ *C* ∈ *R*_*F*_(Π), if there exists *B*_0_→_∧_ *C*_0_ ∈ *R*_*F*_(Π)∖{*B*→_∧_ *C*} such that *B*_0_→_∧_ *C*_0_⇒*B*→_∧_ *C*, then *B*→_∧_ *C* is said to be redundant in *R*_*F*_(Π); otherwise, *B*→_∧_ *C* is said to be nonredundant in *R*_*F*_(Π). One denotes by *R*_F_*(Π) the set of all the nonredundant decision rules in *R*_*F*_(Π).

Thus, for any *B*→_∧_ *C* ∈ *R*_*F*_(Π), one concludes that each object having all the conditional attributes in *B* also has all the decision attributes in *C*. More specifically, if *B* = {*b*_1_, *b*_2_,…, *b*_*s*_} and *C* = {*c*_1_, *c*_2_,…, *c*_*t*_}, then *B*→_∧_ *C* means the following: “if *b*_1_∧*b*_2_∧⋯∧*b*_*s*_, then *c*_1_∧*c*_2_∧⋯∧*c*_*t*_,” where ∧ denotes logical conjunction operator. Moreover, it is easy to observe that decision rules, ∨-rules, and ∨-∧ mixed rules are different from each other in terms of their logical reasoning methodologies.

The following example is used to show that there does not exist inclusion relationship among decision rules, ∨-rules, and ∨-∧ mixed rules. That is, we need to confirm three statements: (1) a decision rule may not be a ∨-rule or ∨-∧ mixed rule; (2) a ∨-rule may not be a decision rule or ∨-∧ mixed rule; (3) a ∨-∧ mixed rule may not be a decision rule or ∨-rule.

**Example 16 .**

**Figure 4:**
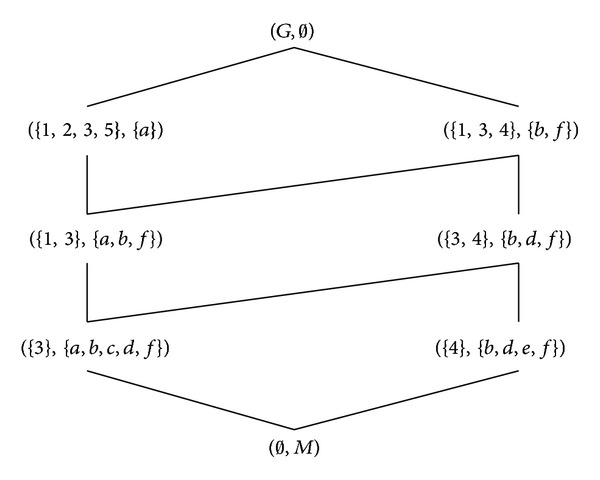
The Hasse diagram of ${\underline{B}}_{F}(G,M,I)$.

Let Π = (*G*, *M*, *I*, *N*, *J*) be the formal decision context in Table 1, where *G* = {1,2, 3,4, 5}, *M* = {*a*, *b*, *c*, *d*, *e*, *f*}, and *N* = {*d*_1_, *d*_2_, *d*_3_}. The formal concept lattice of (*G*, *M*, *I*) is shown in Figure 4 and that of (*G*, *N*, *J*) can be found in Figure 2.

According to the algorithm in [28] (interested readers can refer to [28] about how to efficiently derive all the nonredundant decision rules from a formal decision context), we can derive the following nonredundant decision rules from Π:

 
*r*_1_′′: if *a*, *b*, and *f*, then *d*_1_ and *d*_2_;
 
*r*_2_′′: if *b*, *d*, *e*, and *f*, then *d*_2_ and *d*_3_;
 
*r*_3_′′: if *b* and *f*, then *d*_2_.

Combining these decision rules with the results obtained in Examples 9 and 13, we conclude that there does not exist inclusion relationship among decision rules, ∨-rules, and ∨-∧ mixed rules.

Moreover, the following example is used to show that there does not exist inference relationship among decision rules, ∨-rules, and ∨-∧ mixed rules, where the inference rule is described as follows [32]:

(12) $$\begin{array}{c} \Omega :\;\frac{B_{1}⟶C_{1},B_{1}\subseteq B_{2},C_{2}\subseteq C_{1}}{B_{2}⟶C_{2}}. \end{array}$$

Note that the negation of a conditional attribute is treated as a new one and it is different from others.

**Example 17 .**

Let Π = (*G*, *M*, *I*, *N*, *J*) be the formal decision context in Table 1, where *G* = {1,2, 3,4, 5}, *M* = {*a*, *b*, *c*, *d*, *e*, *f*}, and *N* = {*d*_1_, *d*_2_, *d*_3_}. Then, according to the discussion in Examples 9, 13, and 16, we have the following:

- the decision rule *r*_1_′′ cannot be implied by the ∨-rules *r*_1_, *r*_2_, *r*_3(1)_, *r*_3(2)_, *r*_3(3)_, *r*_3(4)_, and *r*_3(5)_ based on the inference rule *Ω*, neither can the ∨-∧ mixed rules *r*_1_′, *r*_2_′, *r*_3_′, and *r*_4_′;
- each of the ∨-rules *r*_1_, *r*_2_, *r*_3(1)_, *r*_3(2)_, *r*_3(3)_, *r*_3(4)_, and *r*_3(5)_ cannot be implied by the decision rules *r*_1_′′, *r*_2_′′, and *r*_3_′′ based on the inference rule *Ω*, neither can the ∨-∧ mixed rules *r*_1_′, *r*_2_′, *r*_3_′, and *r*_4_′;
- each of the ∨-∧ mixed rules *r*_1_′, *r*_2_′, and *r*_4_′ cannot be implied by the decision rules *r*_1_′′, *r*_2_′′, and *r*_3_′′ based on the inference rule *Ω*, neither can the ∨-rules *r*_1_, *r*_2_, *r*_3(1)_, *r*_3(2)_, *r*_3(3)_, *r*_3(4)_, and *r*_3(5)_.

## 5. Experiments

Although, according to the discussion in Section 4, there does not exist inclusion or inference relationships among decision rules, ∨-rules, and ∨-∧ mixed rules, it is still necessary to conduct some experiments to compare the proposed rule acquisition algorithms with the existing one in [27] in terms of the running efficiency.

In the experiments, eight real-life databases, including Bacteria [43], Zoo [44], Breast Tissue [44], Acute Inflammations [44], Servo [44], Wine [44], Balance Scale [44], and Car Evaluation [44], are analyzed to achieve the task of comparing the running efficiency. The detailed information about the eight chosen real-life databases is shown in Table 2.

For each of the chosen databases, we took the classification attribute(s) as the decision attribute(s) and other attributes as the conditional attributes. Then, the scaling approach [2] was used to transform the eight chosen databases into formal decision contexts. More specifically, discrete (but not Boolean) or continuous attributes were converted into Boolean ones. The detailed information about the conversion is listed in Table 3, where “/” means “taking no action” and “trisection” means “classifying the values of each continuous attribute, from small to large, into three pairwise disjoint intervals whose lengths are the same.” We denote by data sets 1, 2, 3, 4, 5, 6, 7, and 8 the formal decision contexts which were obtained by applying the scaling approach (exactly, nominal scale and/or ordinal scale) to the eight chosen databases.

In the experiments, we still denote the proposed rule acquisition algorithms by Algorithms 1 and 2 (see Sections 3.1 and 3.2 for details) and the existing one in [27] by Algorithm  3. Then, Algorithms 1, 2, and 3 were applied to data sets 1, 2, 3, 4, 5, 6, 7, and 8. The corresponding running time is reported in Table 4 in which “Size” is the Cartesian product of the object set, conditional attribute set, and decision attribute set of the concerned formal decision context and *R*_O_*(Π), *R*_P_*(Π), and *R*_F_*(Π) are the nonredundant ∨-rules, ∨-∧ mixed rules, and decision rules, respectively. It can be seen from Table 4 that, by the running time, Algorithms 1, 2, and  3 are all acceptable, and which one being more efficient than another seems to be dependent on the given databases including density and scaling approach.

## 6. Conclusion and Future Work

Rule acquisition is one of the main purposes in the analysis of formal decision contexts. Although there have been several types of rules in formal decision contexts such as decision rules, decision implications, and granular rules, these rules are all ∧-ones since they have the following form: “if conditions 1,2,…, and *m* hold, then decisions hold.” In order to enrich the existing rule acquisition theory in formal decision contexts, we have proposed two new types of rules, called ∨-rules and ∨-∧ mixed rules, based on formal, object-oriented, and property-oriented concept lattices. Moreover, a comparison of ∨-rules, ∨-∧ mixed rules, and ∧-rules has been made from the perspectives of inclusion and inference relationships. Finally, some numerical experiments have been conducted to compare the proposed rule acquisition algorithms with the existing one in terms of the running efficiency.

From the point of view of real applications, the results obtained in this paper need to be further extended to the cases of fuzzy formal decision contexts [45], incomplete formal decision contexts [29], and real formal decision contexts [30, 31] since in the real world the relationship between some objects and attributes of a formal decision context may be fuzzy-valued, interval-valued, or even real-valued. This issue will be discussed in our future work.

## Figures and Tables

**Algorithm 1:**
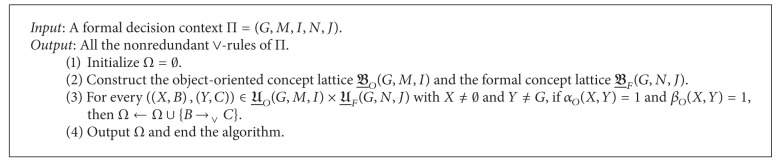
Deriving the nonredundant ∨-rules from a formal decision context.

**Algorithm 2:**
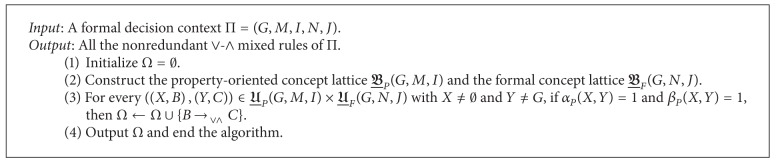
Deriving the nonredundant ∨-∧ mixed rules from a formal decision context.

**Table 1:** A formal decision context Π = (*G*, *M*, *I*, *N*, *J*).

*U*	*a*	*b*	*c*	*d*	*e*	*f*	*d*_1_	*d*_2_	*d*_3_
1	1	1	0	0	0	1	1	1	0
2	1	0	0	0	0	0	0	1	1
3	1	1	1	1	0	1	1	1	0
4	0	1	0	1	1	1	0	1	1
5	1	0	0	0	0	0	1	0	0

**Table 2:** The detailed information about the eight chosen databases in the experiments.

Database	Instances	Classification attribute(s)	Other attributes
Bacteria	17	1 (discrete, 6 values)	16 (Boolean)
Zoo	101	1 (discrete, 7 values)	15 (Boolean), 1 (discrete but not Boolean)
Breast Tissue	106	1 (discrete, 6 values)	9 (continuous)
Acute Inflammations	120	2 (Boolean)	5 (Boolean), 1 (continuous)
Servo	167	1 (continuous)	4 (discrete but not Boolean)
Wine	178	1 (discrete, 3 values)	13 (continuous)
Balance Scale	625	1 (discrete, 3 values)	4 (discrete but not Boolean)
Car Evaluation	1,728	1 (discrete, 4 values)	6 (discrete but not Boolean)

**Table 3:** Converting the eight chosen databases into formal decision contexts.

Database	Data preprocessing		Scaling	
	Conditional attributes	Decision attributes	Conditional attributes	Decision attributes
Bacteria	/	/	/	Nominal scale
Zoo	/	/	Nominal scale for 13th	Nominal scale
Breast Tissue	Trisection	/	Ordinal scale	Nominal scale
Acute Inflammations	Trisection for 1st	/	Ordinal scale for 1st	/
Servo	/	Trisection	Nominal scale	Ordinal scale
Wine	Trisection	/	Ordinal scale	Nominal scale
Balance Scale	/	/	Nominal scale	Nominal scale
Car Evaluation	/	/	Nominal scale	Nominal scale

**Table 4:** A contrast between the proposed rule acquisition algorithms and the existing one in terms of the running time.

Database	Size	Number of rules			Running time(s)		
		*R*_O_*(Π)	*R*_P_*(Π)	*R*_F_*(Π)	Algorithm 1	Algorithm 2	Algorithm 3 [27]
Data set 1	17 × 16 × 6	2	4	6	0.14	0.19	0.08
Data set 2	101 × 21 × 7	3	9	9	41.57	39.81	1.13
Data set 3	106 × 26 × 6	2	0	5	0.13	0.11	0.06
Data set 4	120 × 8 × 2	4	3	5	0.06	0.05	0.11
Data set 5	167 × 19 × 3	167	23	27	11585.91	13198.84	1.66
Data set 6	178 × 39 × 3	3	17	30	4865.34	5088.11	23.12
Data set 7	625 × 20 × 3	2	5	3	48.88	73.34	136.25
Data set 8	1,728 × 21 × 4	1	3	1	430.41	522.78	1981.81
